# Effect of real-time ultrasound imaging for biofeedback on trunk muscle contraction in healthy subjects: a preliminary study

**DOI:** 10.1186/s12891-021-04006-0

**Published:** 2021-02-05

**Authors:** Shanshan Lin, Bo Zhu, Yiyi Zheng, Guozhi Huang, Qi Zeng, Chuhuai Wang

**Affiliations:** 1 Department of Rehabilitation Medicine, The First Affiliated Hospital, Sun Yat-sen University, Guangzhou, 510080 China; 2 Department of Hepatobiliary Surgery, The Second Affiliated Hospital, Guangzhou Medical University, Guangzhou, 510260 China; 3 Department of Rehabilitation Medicine, Zhujiang Hospital, Southern Medical University, Guangzhou, 510282 China

**Keywords:** Multifidus, Transversus abdominis, Ultrasound imaging, Biofeedback

## Abstract

**Background:**

Real-time ultrasound imaging (RUSI) has been increasingly used as a form of biofeedback when instructing and re-training muscle contraction. However, the effectiveness of the RUSI on a single sustained contraction of the lumbar multifidus (LM) and transversus abdominis (TrA) has rarely been reported. This preliminary study aimed to determine if the use of RUSI, as visual biofeedback, could enhance the ability of activation and continuous contraction of the trunk muscles including LM and TrA.

**Methods:**

Forty healthy individuals were included and randomly assigned into the experimental group and control group. All subjects performed a preferential activation of the LM and/or TrA (maintained the constraction of LM and/or TrA for 30 s and then relaxed for 2 min), while those in the experimental group also received visual feedback provided by RUSI. The thickness of LM and/or TrA at rest and during contraction (T_c-max_, T_15s_, and T_30s_) were extracted and recorded. The experiment was repeated three times.

**Results:**

No significant differences were found in the thickness of LM at rest (*P* > 0.999), T_c-max_ (P > 0.999), and T_15s_ (*P* = 0.414) between the two groups. However, the ability to recruit LM muscle contraction differed between groups at T_30s_ (*P* = 0.006), with subjects in the experimental group that received visual ultrasound biofeedback maintaining a relative maximum contraction. Besides, no significant differences were found in the TrA muscle thickness at rest (*P* > 0.999) and T_c-max_ (*P* > 0.999) between the two groups. However, significant differences of contraction thickness were found at T_15s_ (*P* = 0.031) and T_30s_ (*P* = 0.010) between the two groups during the Abdominal Drawing-in Maneuver (ADIM), with greater TrA muscle contraction thickness in the experimental group.

**Conclusions:**

RUSI can be used to provide visual biofeedback, which can promote continuous contraction, and improve the ability to activate the LM and TrA muscles in healthy subjects.

## Background

The lumbar multifidus (LM) and transversus abdominis (TrA) are deep trunk muscles that play crucial roles in the segmental stabilization of the lumbar spine [1, 2]. Defects in these local muscles, such as atrophy, fat infiltration and/or decrease in activation, make preferential activation delay and anticipatory postural adjustment difficult, probably resulting in damage to spinal structure and function [3, 4].

As previously reported, LM and TrA, which were core stabilizing muscles and acted like a corset of the trunk maintaining the spinal segment’s neutral zone, were helpful to relieve low back pain (LBP) and prevent its recurrence by maintaining segmental stabilization and stiffness once spinal stability was threatened [5–8].

The rehabilitative strategies, such as core stabilizing exercise and sling exercise training, strengthened local muscles around the low back, and were effective in reducing low back pain and alleviating dysfunction caused by lumbopelvic instability [9–11]. In clinical work, it was necessary to evaluate muscle function during different exercises to generate efficient rehabilitation approaches. In order to provide real-time, accurate and precise information on specific functional tasks in rehabilitation intervention, reliable and sensitive measurement is in great need. However, it appeared to be hard to measure the function of TrA and LM muscles, whose preferentially activation is particularly difficult, causing the dilemma in curing LBP [3].

Real-time ultrasound imaging (RUSI) has been identified as a noninvasive method to document morphological properties and contraction thickness of muscles. It has been used more commonly both in the clinical and research process [12, 13]. The reliability of ultrasound detection methods in quantifying muscle thickness and contraction has been previously demonstrated [14]. Compared with magnetic resonance imaging and electromyography, RUSI was not only an efficient measurement of the muscle morphology but also an effective biofeedback method to selectively activation of TrA and lumbar multifidus [15–19]. However, the appropriateness and purpose of using RUSI as a biofeedback tool, the timing and mode of biofeedback, as well as the determination of which subgroups of LBP individuals that would obtain most benefit are not fully understood. In particular, there has been little research on the effects of real-time ultrasound feedback on the performance and contraction efficiency of the multifidus and TrA during a single continuous activation training.

Given this, the study aimed to explore whether real-time ultrasound feedback technology could improve or enhance the performance and contraction endurance of the multifidus and TrA in healthy adults during a single continuous preferential activation.

## Methods

### Participants

Forty healthy subjects participated in this study. Inclusion criteria for the current study were: (a) ages between 20 and 45 years; (b) no history of LBP or lower extremity pain in the last 6 months; (c) able to perform the improved Biering-Sorensen test (BST) in the prone position, and Abdominal Drawing-in Maneuver (ADIM) in the supine position for more than 30s; (d) right-handed; and (e) body mass index (BMI) within ±10% of international normal range (the value of BMI 18.5–24.9, indicating normal range). Exclusion criteria were: LBP patients; a history of abdominal or spinal surgery; previous experience of real-time ultrasound imaging on trunk muscles; any medical condition affecting the spine (eg, ankylosing spondylitis, scoliosis, rheumatoid arthritis, osteoporosis, systemic disease, and severe neurological disorders), or pregnancy. All subjects reviewed and signed informed consent that was approved by the Institutional Research Ethics Committee of the First Affiliated Hospital, Sun Yat-sen University. We confirmed that all methods were performed following the ethical standards of the Declaration of Helsinki.

The healthy volunteers were recruited from posted notices and social network platform, both genders were included and were randomly divided into one of two groups. Group 1 (real-time ultrasound visual feedback group, experimental group, Exp) contained 16 females and 4 males and group 2 (verbal instructed group, control group, CG) contained 15 females and 5 males.

### Procedures

Images of the LM and TrA were acquired in B-mode with a portable ultrasound machine (SonoSite M-Turbo, Seattle, USA) with a 2-5 MHz curvilinear-array (for LM) or 6-13 MHz linear-array transducer (for TrA), automatically adjusted by the scanning depth.

All participants were firstly given a verbal explanation regarding the purpose and operation procedure of the experiment and the anatomical structure and function of the multifidus and abdominal muscles before the test. Besides, the locations, the morphological and structural changes of the TrA and LM during contraction were shown and identified using ultrasound imaging in the experimental group. During contraction, subjects in the experimental group were required to watch the real-time ultrasound imaging and maintain continuous contraction of the LM or TrA with maximum effort.

Image acquisition for each condition and each time point (T_rest_, T_c-max_, T_c-15s_, T_c-30s_) was repeated three times. To maximize time efficiency, one operator recorded the time and saved the image, whereas the other operator positioned the transducer and optimized the quality of the image. Both operators undergone extensive training in the area of RUSI and had at least 5 years of experience with RUSI in a musculoskeletal setting. To avoid potential fatigue associated with the detection sequence, the muscles (TrA and LM) being tested and imaged were randomly assigned.

### Assessment of LM thickness

Images of the right LM at rest and during maximum isometric contraction (MIC) were acquired following procedures outlined by Zhang et al. [20] using ultrasound. Participants underwent measurements in randomly-assigned order. Participants were firstly in a prone lying position, with a pillow placed under the abdomen to diminish the lumbar lordosis. The lumbar spinous processes from L1 to S1 were palpated and marked on the skin with an indelible marker, which was then identified by real-time ultrasound.

**Fig. 1:**
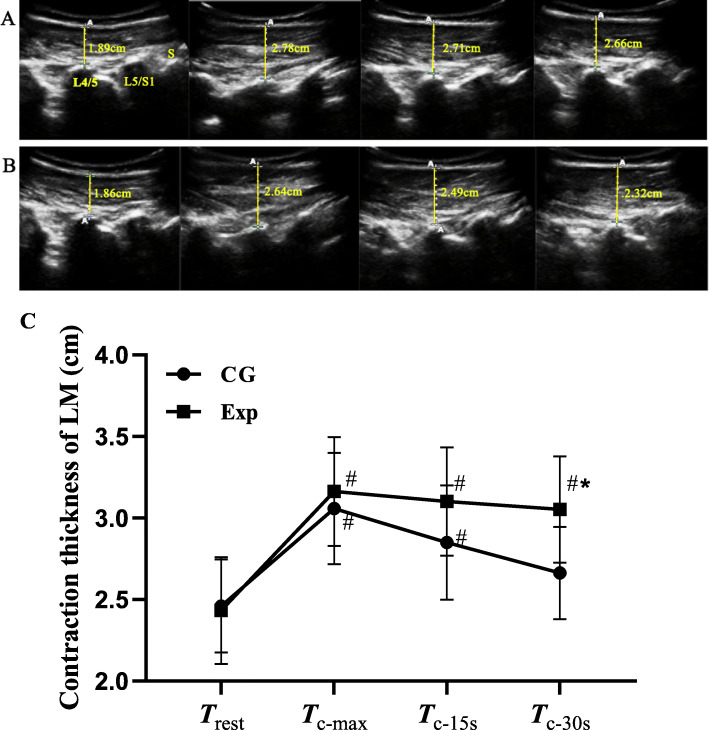
Thickness of LM monitored under ultrasound. **a** Changes of the LM thickness of a subject in the Exp group, (**b**) changes of the LM thickness of a subject in the control group, and (**c**) changes of the LM thickness in the two groups over time. ^**#**^ Compared with the resting state, *P* < 0.05; ^*^ Compared with the control group, *P* < 0.05

The thickness of the right LM was imaged in a longitudinal section at the L4–5 level. Three separate resting ultrasound images were collected immediately after exhalation. To avoid affecting the muscle shape, the operator must be careful not to compress the skin with the transducer. Then, subjects were asked to maintain back muscle contractions for 30 s, and performed three times with a two-minute interval between tasks. To obtain the maximum isometric contraction data, subjects were positioned in the standard prone position with the upper limbs positioned overhead, shoulders abducted to approximately 120°. Subjects were then asked to lift their head, trunk, and upper extremities with maximum effort, which had been used previously to activate the contraction of LM [20–22]. Oral encouragement was given to the individuals in the control group during MIC. Besides the verbal instruction, subjects in the experimental group were required to watch the real-time ultrasound imaging and maintain continuous contraction of the LM with maximum effort. Image acquisitions were collected at three time points (T_c-max_, T_c-15s_, T_c-30s_) during contraction, and the experiment was repeated three times. A single practice and two preliminary acquisitions were executed before images being recorded. Linear measurements of the LM thickness were taken in all subjects using on-screen calipers from the superior border of the LM to the tip of the L4–5 zygapophyseal joint (Fig. 1a, b).

### Assessment of TrA thickness

Images of the right TrA muscle were acquired at rest and during the ADIM maneuver [23, 24] with subjects in a supine hook-lying position and their arms crossed over the chest. US images were collected with the transducer placed transversely just along the midaxillary line at the level of the umbilicus, in which the middle of the TrA muscle belly was positioned within the field of view [25]. All images were obtained at the end of exhalation to avoid the influence of respiration.

**Fig. 2:**
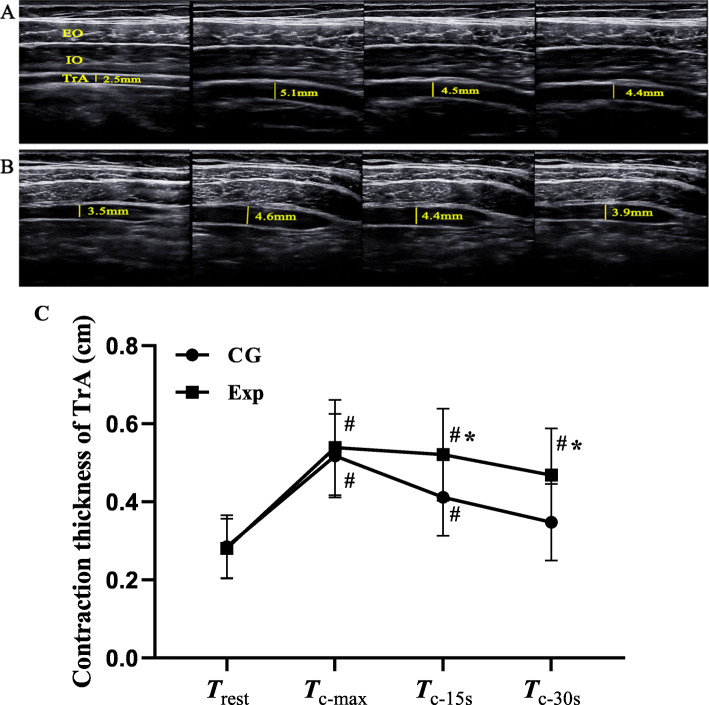
Thickness of TrA monitored under ultrasound. **a** Changes of the TrA thickness of a subject in the Exp group, (**b**) changes of the TrA thickness of a subject in the control group, and (**c**) changes of the TrA thickness in the two groups over time. ^**#**^ Compared with the resting state, *P* < 0.05; ^*^ Compared with the control group, *P* < 0.05

The ADIM is a fundamental motor control training used to preferentially activate the TrA muscle contraction in comparison with the more superficial abdominal muscles [25]. In this study, the ADIM was used to assess the altered muscle thickness associated with a voluntary contraction of the TrA muscle. To perform the ADIM, participants were instructed to “draw-in your umbilicus toward the spine without moving back or pelvis, while comfortably breathing in and out.” The angle of the transducer was adjusted properly to collect the best quality images while keeping the transducer perpendicular to the surface of the skin in the same position. During the ADIM, the participants in the experimental group were asked to view the changes of muscle thickness on the ultrasound monitor as visual feedback in an attempt to maximize a preferential and continuous TrA contraction. Image acquisitions were obtained at three time points (T_c-max_, T_c-15s_, T_c-30s_) during ADIM, and were performed 3 times separately with a 2mins interval between each ADIM task. The thickness of the TrA muscle was assessed from the superior fascial border to the inferior fascial border in centimeters (Fig. 2a, b).

### Statistical analysis

Statistical analysis was performed using SPSS 22.0 (SPSS Inc. Chicago, IL, USA). Shapiro-Wilk test was used to identify the normality of data distribution. Baseline demographic characteristics such as age, weight, height, and BMI were reported with descriptive statistics (mean ± SD) and were compared between groups with unpaired t-test. Separate 2-by-4 repeated measures analyses of variance (ANOVAs) were used to examine intervention effects (dependent variables), with the group (control or experimental) as between-subject variable and time (T_rest_, T_c-max_, T_c-15s_, T_c-30s_) as the within-subject variable. The LM and TrA thickness at each time point was reported as a mean with the standard deviation of each group. Post-hoc tests for multiple comparisons with Bonferroni adjustments were adopted when a significant interaction effect existed. The significance level was set at a priori alpha level of 0.05.

## Results

The demographic characteristics of the subjects were provided in Table 1. All subjects satisfactorily cooperated and completed the LM and TrA contraction tasks without verbal complaints of pain. Statistical analysis results showed that there were no significant differences in demographic data between the two groups.

**Table 1:** Participant demographics (mean ± SD)

group	Participants (n)	Gender (F/M)	Age (years)	Weight (kg)	height(m)	BMI (kg/m^2^)
Exp	20	16/4	25.95 ± 2.89	55.65 ± 8.45	1.62 ± 0.06	21.08 ± 1.85
CG	20	15/5	26.60 ± 2.58	56.25 ± 9.14	1.63 ± 0.06	21.02 ± 2.00
*P*		0.705	0.458	0.803	0.601	0.922

*Exp* Experimental group, *CG* Control group

Results of the ANOVA for intervention effects on LM thickness showed significant interaction effect between the factors of time and group (*F* = 3.137, *P* = 0.027), also statistically significant main effects due to time (*F* = 31.45, *P* <  0.001) and group (*F* = 12.34, *P* <  0.001). The results showed that no significant differences were found in the thickness of LM at rest, T_c-max_ and T_15s_ between groups (*P* > 0.999, P > 0.999, and *P* > 0.414, respectively). However, the ability to recruit LM muscle contraction differed at T_30s_ between groups, with subjects in the experimental group that obtained visual ultrasound biofeedback maintaining a relative maximum contraction compared to the CG group (*P* = 0.006). At the time point of T_c-max_, T_15s_, and T_30s_, the results of the intra-group comparison showed that LM contraction thickness in the Exp group was superior to T_rest_ (*P* <  0.001). While, in the CG group, the thickness of LM had no significant statistical significance in T_30s_ compared with T_rest_ (*P* > 0.999). (Table 2 and Fig.1c).

**Table 2:** Comparison of LM thickness at different time points between two groups (mean ± SD, cm)

Time point	Exp (*n* = 20)	CG (*n* = 20)
T_rest_	2.432 ± 0.328	2.460 ± 0.285
T_c-max_	3.162 ± 0.334^**#**^	3.058 ± 0.342^**#**^
T_15s_	3.101 ± 0.332^**#**^	2.849 ± 0.350^**#**^
T_30s_	3.052 ± 0.326^**#**,*^	2.662 ± 0.282
*F*_group_ */ P* value		12.34 / < 0.001
*F*_time_ */ P* value		31.45 / < 0.001
*F*_group*time_ */ P* value		3.137 / 0.027

NOTE: *Exp* Experimental group, *CG* Control group. ^**#**^ Compared with the resting state, *P* <  0.05;

^*^ Compared with the control group, *P* <  0.05

Results of the ANOVA for intervention effects on TrA thickness showed significant interaction effect between the factors of time and group (*F* = 3.583, *P* = 0.015) and statistically significant main effects due to time (*F* = 40.54, *P* < 0.001) and group (*F* = 14.01, *P* < 0.001). Post-hoc analysis showed that there were no significant differences in TrA thickness between the two groups at T_rest_ and T_c-max_ (*P* > 0.999 and *P* > 0.999, respectively). While, at 15 s and 30s of continuous contraction, TrA contracted thickness in the Exp group was significantly better than that in the CG group (*P* = 0.031 and *P* = 0.010, respectively). At the time point of T_c-max_, T_15s_, and T_30s_, the results of the intra-group comparison showed that TrA contraction thickness in the Exp group was superior to T_rest_ (*P* < 0.001). While, in the CG group, the thickness of TrA had no significant statistical significance in T_30s_ compared with T_rest_ (*P* > 0.999). (Table 3 and Fig.2c).

**Table 3:** Comparison of TrA thickness at different time points between two groups (mean ± SD, cm)

Time point	Exp (*n* = 20)	CG (*n* = 20)
T_rest_	0.281 ± 0.076	0.285 ± 0.081
T_c-max_	0.539 ± 0.122^**#**^	0.518 ± 0.103^**#**^
T_15s_	0.521 ± 0.118^**#**,*^	0.412 ± 0.099^**#**^
T_30s_	0.468 ± 0.120^**#**,*^	0.348 ± 0.098
*F*_group_ */ P* value		14.01 / < 0.001
*F*_time_ */ P* value		40.54 / < 0.001
*F*_group*time_ */ P* value		3.583 / 0.015

NOTE: *Exp* Experimental group, *CG* Control group. ^**#**^ Compared with the resting state, *P* < 0.05;

^*^ Compared with the control group, *P* < 0.05

## Discussion

The coordinated systems of neural components, passive spinal-column anatomy and spinal muscles (global and local muscles) contribute to the spinal stability [6]. Global muscles such as the erector spinae and rectus abdominis are the prime movers of the spine, while local stabilizing muscles such as TrA and LM are thought to provide segmental stability of the spine. Coordination between prime movers and local stabilizers has been regarded as an important neuromuscular component of maintaining a non-painful low back [6].

Individuals with LBP exhibit altered neuromuscular control, amyotrophy, or a reduction in anticipatory activation of the local muscles, resulting in lumbar instability [26, 27]. Preferentially activating the selected trunk muscles such as TrA and LM muscles appears to be particularly difficult in LBP patients [28, 29]. Therefore,rehabilitation programs for enhancing the ability of LBP subjects to re-establish appropriate sensory-motor loops and promoting activation of local stabilizingmusculature of the spine have become a promising treatment in LBP individuals [30].

Researchers and clinicians have been using RUSI to provide visual feedback during training to enhance mastery of the ADIM and LM muscle performance in individuals with LBP. Van et al. [31] demonstrated that subjects who received the clinical instruction and visual feedback from RUSI obtained greater improvements in the preferential activation of the LM. Henry et al. [32] reported that using RUSI as visual feedback reduced the number of trials needed for subjects without LBP to perform the ADIM correctly. However, Teyhen et al. [33] reported a negative result for the use of RUSI to enhance ADIM performance in a group of LBP individuals. The appropriateness and effectiveness of using RUSI as a biofeedback tool are not clearly understood. The effect of pretraining, as well as the timing, type and amount of feedback, might be the possible factors affecting the effectiveness of the biofeedback [34].

The current study evaluated the contraction efficiency of the TrA and LM muscles in healthy subjects at rest and during ADIM or BST tasks at a single session by providing verbal instruction alone versus verbal instruction plus visual real-time ultrasound feedback. The results of the present study indicated that real-time ultrasound imaging did not affect the maximum muscle contraction thickness of the TrA or LM at the beginning. However, there were significant differences in contraction thickness between the two groups with the prolonging of time (Tables 2 and 3). The results demonstrated that subjects in the experimental group that received verbal instruction plus visual biofeedback improved their contraction thickness and performed consistently better than subjects that received only verbal instruction at a relative long-time ADIM and LM isometric contraction. The results showed that real-time ultrasonic feedback could increase the motor control of the TrA and LM muscle during the continuous contraction in healthy adults.

Partner’s study [35] observed the percent change in LM-muscle thickness and TrA preferential activation ratio in LBP patients before and after a single session of exercise (completed both exercises for 15 repetitions each with 5 s duration) with or without biofeedback. The results showed that no changes were observed in the percentage of LM-muscle thickness after exercise, indicating that the addition of visual and tactile biofeedback during a 5-s exercise did not result in a statistically significant difference, which were partly similar to the current findings. In our study, the results indicated no difference in the thickness of LM and TrA at rest and T_c-max_ between the two groups, even at T_15s_ for LM thickness. However, significant differences of contraction thickness were found at T_15s_ and T_30s_ for TrA thickness, and T_30s_ for LM thickness between the two groups during the continuous isometric contraction, with greater muscle contraction thickness in the experimental group. The results of our study demonstrated that continuous RUSI feedback during isometric contraction would be useful to improve the performance and efficiency of muscle contraction.

### Limitations

This study had several limitations. Firstly, the current study contained only a single examine session, we were unable to conclude if multiple training sessions or voluntary activation of the abdominal and multifidus muscles would provide additional support to the lumbar spine. Secondly, only ultrasound imaging was used in the current study to measure muscle thickness, which only helped observe muscle activation through thickness changes but could not provide other information such as the onset or timing of muscle activation. Furthermore, we did not incorporate voluntary activation of the ADIM and LM muscle at various positions such as sitting and standing, the current testing position might not be sensitive enough to detect changes in muscle thickness. Otherwise, the position we chosen for contracting the LM muscle in healthy subjects appears a little difficult to LBP patients, which need further verification. Finally, only healthy subjects were recruited in this study, the relationship between the improvement of muscle performance and clinical outcomes in LBP individuals via RUSI required further investigation. The above issues required future studies that would assess the appropriateness of visual feedback provided by RUSI for motor learning and performance in LBP individuals.

## Conclusions

The current results might provide a preliminary evidence to support the use of RUSI as a visual biofeedback technique that could lead to effective and sustained contraction of TrA and LM during training. RUSI could be a useful feedback tool to enhance motor learning and performance, which can promote continuous contraction efficiency, improve the activation ability of the LM and TrA muscles in healthy subjects. Further research is required to determine the effectiveness of RUSI biofeedback for the re-education of other muscles and LBP individuals.

## Data Availability

The datasets used and/or analyzed during the current study are available from the corresponding author on reasonable request.

## Abbreviations

LM: Lumbar multifidus
TrA: Transversus abdominis
RUSI: Real-time ultrasound imaging
ADIM: Abdominal drawing-in maneuver
LBP: Low back pain
BST: Biering-sorensen test
MIC: Maximum isometric contraction
T_c-max_: The time point of maximum isometric contraction
