# Correction to: Effect of real-time ultrasound imaging for biofeedback on trunk muscle contraction in healthy subjects: a preliminary study

**DOI:** 10.1186/s12891-021-04429-9

**Published:** 2021-06-30

**Authors:** Shanshan Lin, Bo Zhu, Yiyi Zheng, Guozhi Huang, Qing Zeng, Chuhuai Wang

**Affiliations:** 1 Department of Rehabilitation Medicine, The First Affiliated Hospital, Sun Yat-sen University, Guangzhou, 510080 China; 2 Department of Hepatobiliary Surgery, The Second Affiliated Hospital, Guangzhou Medical University, Guangzhou, 510260 China; 3 Department of Rehabilitation Medicine, Zhujiang Hospital, Southern Medical University, Guangzhou, 510282 China

**Correction to: BMC Musculoskelet Disord 22, 142 (2021)**

**https://doi.org/10.1186/s12891-021-04006-0**

Following the publication of the original article [1] the authors noticed that the author's name of "Qing Zeng" was misspelled as "Qi Zeng" in the published version.

The original article [1] has been updated.
